# Early Postnatal Exposure to a Low Dose of Decabromodiphenyl Ether Affects Expression of Androgen and Thyroid Hormone Receptor-Alpha and Its Splicing Variants in Mouse Sertoli Cells

**DOI:** 10.1371/journal.pone.0114487

**Published:** 2014-12-05

**Authors:** Hidenobu Miyaso, Noriko Nakamura, Munekazu Naito, Shuichi Hirai, Yoshiharu Matsuno, Masahiro Itoh, Chisato Mori

**Affiliations:** 1 Center for Preventive Medical Sciences, Chiba University, Chiba, Japan; 2 Department of Bioenvironmental Medicine, Graduate School of Medicine, Chiba University, Chiba, Japan; 3 Department of Pharmacology, Physiology and Toxicology, Marshall University, Joan C. Edwards School of Medicine, Huntington, United States of America; 4 Department of Anatomy, Aichi Medical University, Aichi, Japan; 5 Department of Anatomy, Tokyo Medical University, Tokyo, Japan; Inserm, France

## Abstract

Decabromodiphenyl ether (decaBDE) adversely affects reproduction and development. Our previous study showed that postnatal exposure to a low dose of decaBDE (0.025 mg/kg body weight/day) by subcutaneous injection on postnatal days (PNDs) 1 through 5 leads to reductions in testicular size and number of Sertoli cells and sperm, while higher dose of decaBDE (2.5 mg/kg body weight/day) had no significant differences about these. In the present study, we examined the molecular mechanism of these effects on mouse testes following postnatal exposure to a low decaBDE dose. We hypothesized that postnatal exposure to decaBDE may alter levels of serum thyroid hormones (THs) and testosterone, or the level of TH receptor alpha (*Thra*) transcripts and its splicing variants and androgen receptor (*Ar*) in Sertoli cells, adversely affecting spermatogenesis. To test this hypothesis, we examined serum TH and testosterone levels and the levels of transcripts of the *Ar, Thra* and its splicing variants, and *Thra* splicing factors (*Hnrnpa1*, *Srsf1*, and *Hnrnph1*) with qPCR in isolated mouse Sertoli cells exposed postnatally to decaBDE (0.025, 0.25, and 2.5 mg/kg). Levels of serum testosterone and transcripts encoding *Ar*, *Thra*, and its variant, *Thra1,* declined significantly in Sertoli cells of mice exposed to 0.025 mg decaBDE/kg. No significant differences in serum TH level or *Thra2, Hnrnph1*, or *Srsf1* transcript levels were observed between control and decaBDE-exposed mice. However, the *Thra1*:*Thra2* and *Hnrnpa1*:*Srsf1* ratios were altered in Sertoli cells of mice exposed to 0.025 mg decaBDE/kg but not in cells exposed to 0.25 or 2.5 mg decaBDE/kg. These results indicate that postnatal exposure to a low dose of decaBDE on PNDs 1 through 5 lowers the testosterone level and the levels of *Ar* and *Thra* transcripts in Sertoli cells, accompanied by an imbalance in the ratios of *Thra* splicing variants, resulting in smaller testicular size and impaired spermatogenesis.

## Introduction

The polybrominated diphenyl ether (PBDE), decabromodiphenyl ether (decaBDE), is widely used as a flame retardant and is a common environmental pollutant. PBDEs have thyroid-disrupting effects due to structural similarities with thyroid hormones (THs) [1]–[3]. DecaBDE also reportedly disrupts TH levels in fish and rodents [4]–[7]. Similar to other studies [8]–[10], we previously showed that decaBDE exhibits male reproductive toxicity in mice. Postnatal exposure of mice to a low dose (0.025 mg/kg body weight) of decaBDE leads to reduced testicular weight, lower numbers of Sertoli cells and elongated spermatids, and reduced sperm count [11]. However, the detailed mechanism underlying these effects remains unclear.

Sertoli cells play a critical role in spermatogenesis by supporting germ cell differentiation in testicular seminiferous tubules through a number of hormones [12]. Several THs (thyroxine [T_4_] and triiodothyronine [T_3_], but especially T_3_) play important roles in regulating Sertoli cell proliferation and maturation by binding to TH receptors (TRs) [13], [14]. Two isoforms in mice have been identified, TRα and TRβ, which are encoded by the genes *Thra* and *Thrb*, respectively [15], [16]. *Thra* has three splicing variants (*Thra1*, *Thra2*, and *Thra3*), whereas *Thrb* has two splicing variants (*Thrb1* and *Thrb2*) [17], [18]. Although Sertoli cells express the TRα isozymes encoded by *Thra1*, *Thra2*, and *Thra3* and the TRβ isozyme encoded by *Thrb1*, binding of the isozyme encoded by *Thra1* to T_3_ is necessary for normal maturation of Sertoli cells [13]. The androgen receptor (AR) is also expressed in Sertoli cells [19], [20]. In Sertoli cells, the AR functions in the maintenance of testicular development, thereby helping to guarantee male fertility [21], [22]. Similar to testosterone, exposure to T_3_ reportedly increases the level of *Ar* transcripts in cultured Sertoli cells [23].

Serine/arginine-rich splicing factor 1 (SRSF1) plays an important role in regulating alternative splicing by associating with heterogeneous nuclear ribonucleoprotein A1 (HNRNPA1) [24], [25]. SRSF1 and HNRNPA1 have opposing actions *in vivo* [26], with HNRNPA1 functioning as a repressor [27]. Thus, alterations in the SRSF1 to HNRNPA1 ratio affect the alternative splicing of genes [25]. SRSF1 and heterogeneous nuclear ribonucleoprotein H1 (HNRNPH1) reportedly regulate the splicing of *Thra2* by binding to purine residues within a *Thra2*-specific 5′ splicing site [28]. Thus, there appears to be an inverse correlation between the *Hnrnpa1*:*Srsf1* and *Thra1*:*Thra2* expression ratios [28].

Based on the above data, we hypothesized that the molecular mechanism underlying the effects resulting from exposure of mice to low doses of decaBDE on postnatal days (PNDs) 1 through 5 involves changes in the normal levels of serum THs and testosterone or transcripts of *Ar*, *Thra* and its splicing variants in Sertoli cells, resulting in the disruption of spermatogenesis. To test this hypothesis, we examined serum TH and testosterone levels and the levels of transcripts encoding *Ar*, *Thra* and its splicing variants, and the levels of transcripts encoding the *Thra2* splicing factors *Hnrnpa1*, *Srsf1*, and *Hnrnph1* in Sertoli cells isolated from testes of mice exposed postnatally to decaBDE.

## Materials and Methods

### Ethics Statement

All experimental procedures were performed in accordance with the Chiba University Guide for the Care and Use of Laboratory Animals. The study and protocol were approved by the Ethics Committee of Chiba University, Graduate School of Medicine (Approval numbers: 26–91 and 25–141). Mice were sacrificed at 12 weeks of age (PND 84) by CO_2_ euthanasia, after which whole blood and testes were collected.

### Animals

Twelve-week-old ICR male (n = 12) and female (n = 12) mice were purchased from Japan SRL (Hamamatsu, Japan) and then mated. The pregnant dams were randomly divided into four groups. After birth, five male pups were selected from each litter. The mice were housed in a temperature-controlled room (24–26°C) under a 12-h light/dark cycle. CLEA rodent chow CE2 (CLEA Japan Inc., Tokyo, Japan) and water were provided *ad libitum*.

### Reagents

DecaBDE was purchased from Wako Pure Chemical Industries (Osaka, Japan). All other reagents were obtained from Sigma-Aldrich (St. Louis, MO, USA), unless otherwise indicated.

### Administration of decaBDE

Mice were administered decaBDE at doses of 0.025, 0.25, or 2.5 mg/kg body weight/day by subcutaneous injection on PNDs 1 through 5, as previously described [11]. Control group mice were injected similarly with corn oil only.

### Measurement of serum TH (T_3_, T_4_, free T_3_, and free T_4_) and testosterone levels

Blood samples were collected by cardiac puncture from control and decaBDE-exposed mice. Serum was prepared by centrifugation of whole blood at 880 g for 20 min at room temperature. The levels of serum T_3_ and T_4_ were measured using rodent triiodothyronine and thyroxine ELISA test kits (Endocrine Technologies Inc., Newark, CA, USA), respectively, according to the manufacturer's instructions. Similarly, the levels of free T_3_ (FT_3_) and free T_4_ (FT_4_) in the serum were measured using free tri-iodothyronine indes and free thyroxine ELISA kits (Cusabio Biotech Co., Wuhan, PR China), respectively, according to the manufacturer's instructions. A rodent testosterone ELISA kit (Endocrine Technologies) was used to measure serum testosterone levels.

### Establishment of primary Sertoli cell culture

Testes from control and decaBDE-exposed mice were resected and the tunica albuginea was removed. The seminiferous tubules were incubated at room temperature for 2 h with gentle agitation in Eagle's minimal essential medium (pH 7.2) (Sigma-Aldrich) containing 0.1% collagenase (Wako Pure Chemical Industries). The suspensions of the control and the decaBDE-exposed mice were removed from agitation and allowed to stand for 5 min to precipitate the seminiferous tubules. The supernatants were discarded and the seminiferous tubules were dispersed gently in PBS containing 1 mM EDTA and placed at room temperature for two-three minutes. The suspensions were centrifuged at 600× *g* for 10 min, and the resulting supernatants were discarded. The tubules were then incubated with 0.25% trypsin (Sigma-Aldrich) for 10 min, after which each suspension was filtered through a nylon mesh (40 µm, Corning Inc., Corning, NY, USA) and centrifuged at 600× *g* for 10 min. The supernatant was discarded and the tubules were washed once with PBS. The tubules were dispersed in Eagle's minimal essential medium (pH 7.2) containing 10% FBS (Gibco, Carlsbad, CA, USA) and 1% penicillin-streptomycin (Sigma-Aldrich), plated on collagen-coated culture dishes (Iwaki, Tokyo, Japan), and incubated at 32.5°C in a 5% CO_2_ atmosphere to allow for growth of Sertoli cells. After 2 h, the medium was replaced and the cells were continuously incubated at 32.5°C in a 5% CO_2_ atmosphere. After 1 week, the Sertoli cells were collected and stored at −80°C until used.

### RNA extraction and real-time PCR (qPCR)

Total RNA was extracted from isolated Sertoli cells using Trizol reagent (Invitrogen, Carlsbad, CA, USA). Reverse-transcription of RNA for real-time PCR was performed with a QuantiTect Reverse Transcription kit (Qiagen, Valencia, CA, USA), according to the manufacturer's protocol. A DNA Engine Opticon (MJ Research Inc, Cambridge, MA, USA) and SYBR Green Real-time PCR Master Mix (Toyobo, Osaka, Japan) were used for real-time PCR, according to the manufacturers' instructions. The primer sequences used for qPCR are shown in Table 1. Briefly, the thermocycling program consisted of one cycle at 95°C for 1 min, 40 cycles of denaturation at 95°C for 15 s, and annealing plus elongation for 1 min at 49.9°C for *Thra*, 68.2°C for *Thra1*, 66.5°C for *Thra2*, 68.2°C for *Ar*, 68.2°C for *Hnrnpa1*, 68.2°C for *Srsf1*, 54.0°C for *Hnrnph1*, or 68.2°C for beta-actin (*Actb*). The level of each mRNA transcript was normalized to that of *Actb* as an internal control.

**Table 1:** Primer pairs used for qPCR.

Gene	Primer sequences	Primer position	Amplified size (bp)	GenBank accession number
*Thra*	For: 5′-GGA TGG AAT TGA AGT GAA TGG AA-3′	414–436	109	NM_178060.3
	Rev: 5′-CCG TTC TTT CTT TTT CGC TTT C-3′	500–522		
*Thra1*	For: 5′- CTG CCT TGC GAA GAC CAG ATC-3′	865–884	307	X07750.1
	Rev: 5′-CGA CTT TCA TGT GGA GGA AG-3′	1,152–1,171 (*Thra1*specific)		
*Thra2*	For: 5′-AAT GGT GGC TTG GGT GTG GT-3′	865–884	364	X07751.1
	Rev: 5′-CCT GAA CAA CAT GCA TTC CGA-3′	1,208–1,228 (*Thra2* specific)		
*Ar*	For: 5′-GCC CCC ATC CAA GAC CTA TCG-3′	71–91	145	NM_013476.3
	Rev: 5′-GCT AGT CTC CTG CCT CTG CTG-3′	195–215		
*Hnrnpa1*	For: 5′-TGG AAG CAA TTT TGG AGG TGG-3′	946–966	155	BC092395.1
	Rev: 5′-GGT TCC GTG GTT TAG CAA AGT-3′	1,080–1,100		
*Srsf1*	For: 5′-CAC TGG TGT CGT GGA GTT TG-3′	566–585	189	BC046773
	Rev: 5′-CTT CTG CTA CGG CTT CTG CT-3′	735–754		
*Hnrnph1*	For: 5′-ATT GCA TAG GTA GCC AAG G-3′	1,396–1,414	119	BC056224.1
	Rev: 5′-CCA TCC ACC ACT CAT ACT AGA C-3′	1,493–1,514		
*Hnrnpf*	For: 5′-GGC ATC TGT GGT GGT TCT TT-3′	226–245	175	NM_133834.2
	Rev: 5′-TGC AGT CGG AGA GGA AGT TT-3′	381–400		
*Actb*	For: 5′-AGA GGG AAA TCG TGC GTG AC-3′	693–712	138	NM_007393.3
	Rev: 5′-CAA TAG TGA TGA CCT GGC CGT-3′	810–830		

### Statistical analysis

Results were evaluated for statistical significance using one-way ANOVA followed by Dunnett's test. All values are expressed as the mean and standard deviation (SD). A *P* value <0.05 was considered to indicate a significant difference.

## Results

### No significant changes were detected in serum TH levels between control and decaBDE-exposed groups

**Figure 1:**
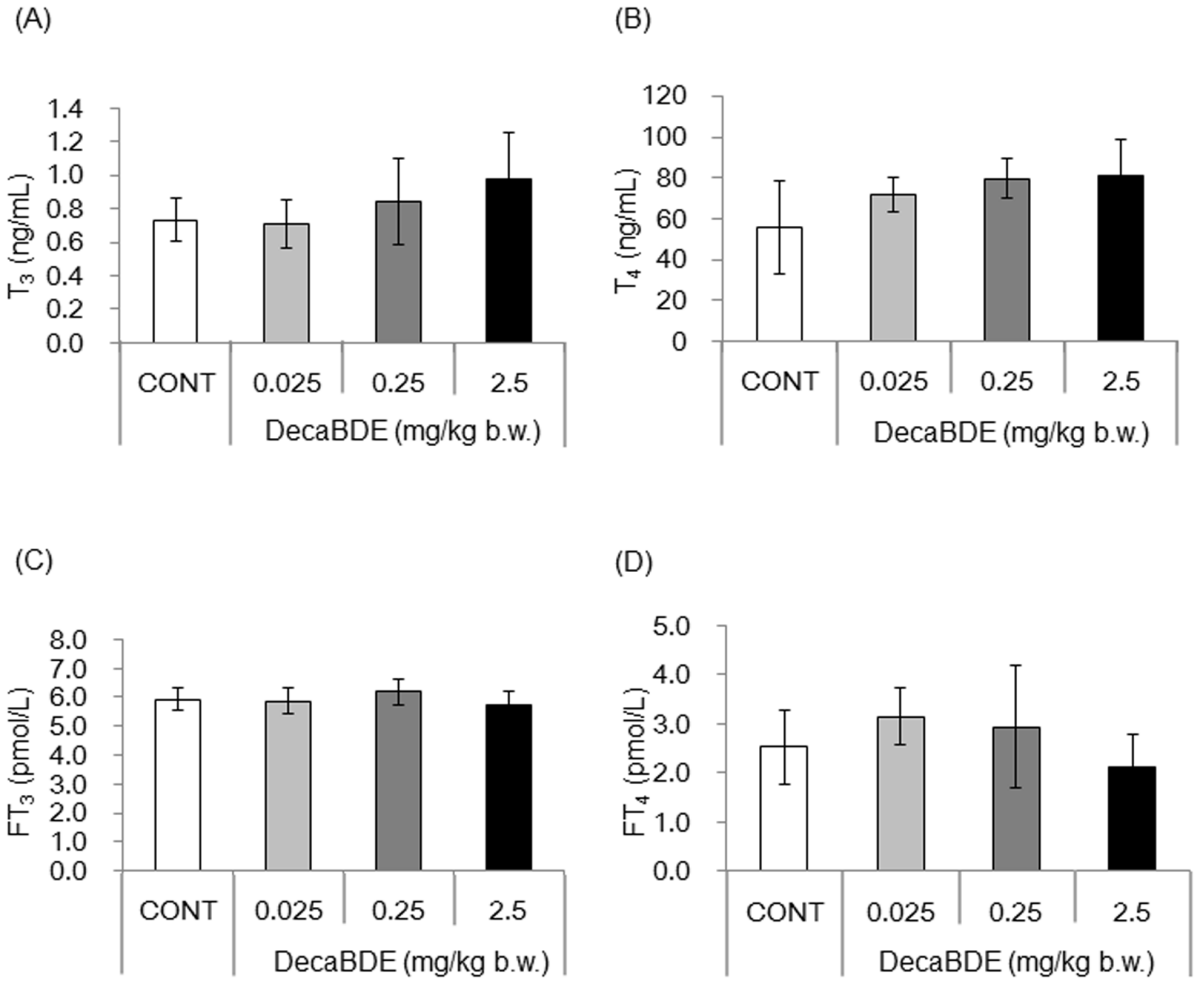
Serum TH levels in control and decaBDE-exposed mice. Total T_3_ (A), total T_4_ (B), FT_3_ (C) and FT_4_ (D) levels in the serum of control and decaBDE-exposed mice were measured by ELISA. Each value is the mean ± SD of 6 samples per group.

To determine whether postnatal exposure to decaBDE disrupts normal TH levels, we measured the concentrations of serum T_3_, T_4_, FT_3_, and FT_4_ by ELISA. Although the serum levels of T_3_ and T_4_ tended to increase in a dose-dependent manner, no significant difference was observed between the control and decaBDE-exposed mice (Fig. 1A, B). In addition, no significant changes in the levels of serum FT_3_ and FT_4_ were observed between the control and decaBDE-exposed mice (Fig. 1C, D).

### Serum testosterone levels were decreased in the 0.025 and 0.25 mg/kg exposed-groups

**Figure 2:**
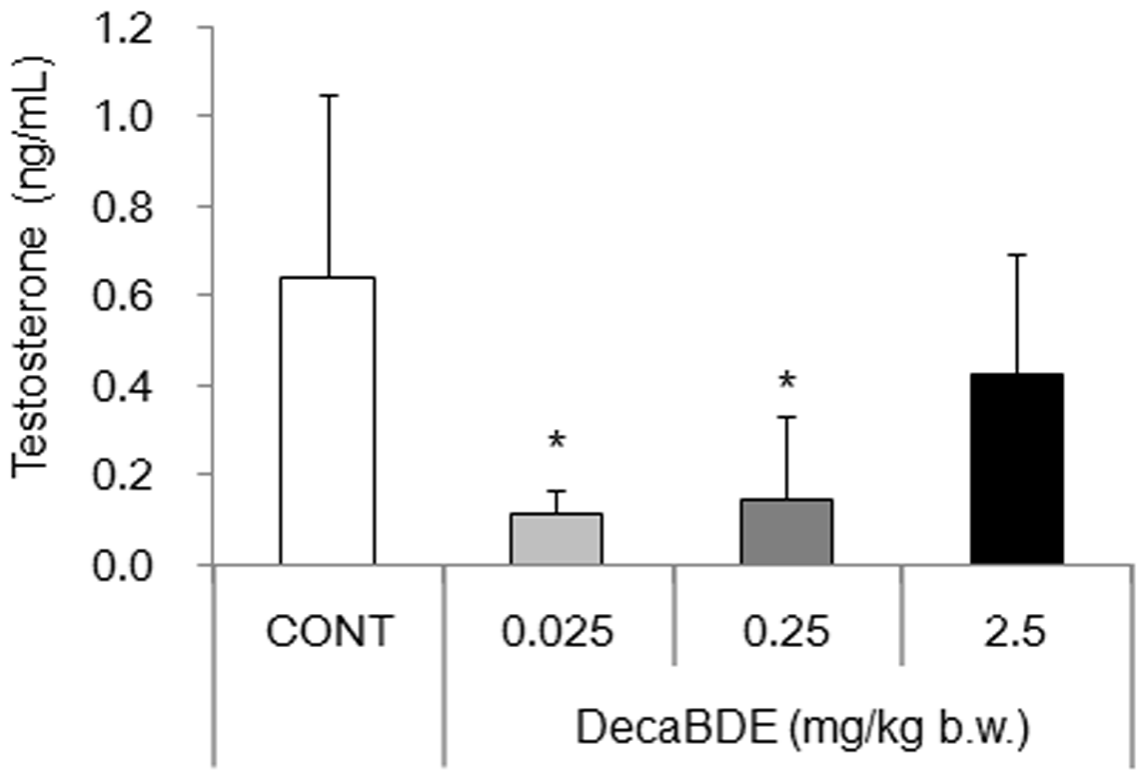
Serum testosterone levels in control and decaBDE-exposed mice. Testosterone levels in the serum of control and decaBDE-exposed mice were measured by ELISA. Each value is the mean ± SD of 6 samples per group. *P<0.05 compared with the control.

The effect of postnatal exposure to decaBDE on serum testosterone levels was evaluated by ELISA. Serum testosterone levels were significantly lower in mice exposed to decaBDE at 0.025 and 0.25 mg/kg than in the controls (Fig. 2). There was no significant difference in testosterone level between the control mice and mice exposed to decaBDE at 2.5 mg/kg.

### Expression levels of *Thra* and its splicing variant (*Thra1*) were reduced in isolated Sertoli cells of mice exposed to 0.025 mg decaBDE/kg

**Figure 3:**
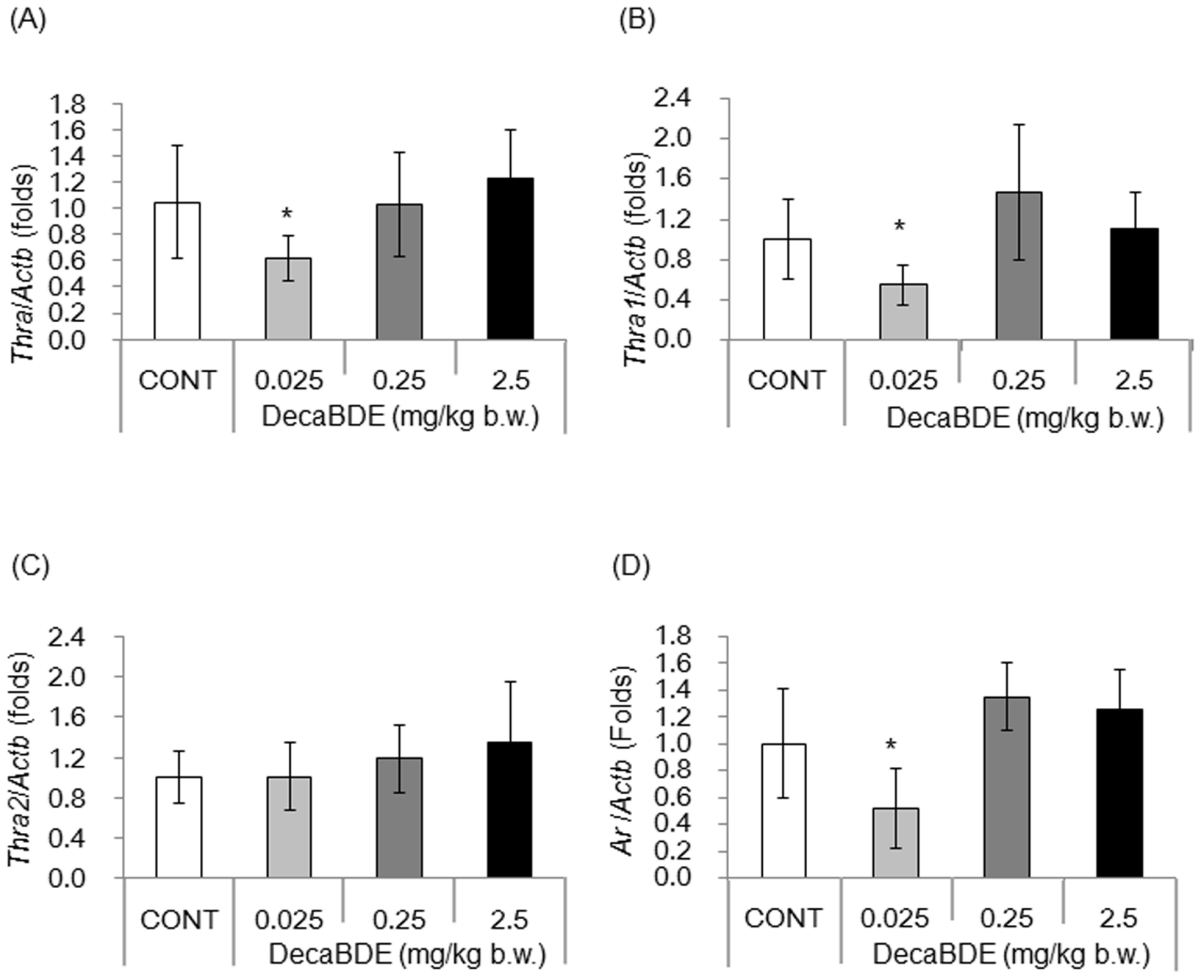
Levels of *Thra* and *Ar* transcripts in isolated Sertoli cells. Levels of transcripts of *Thra* (A), *Thra1* (B), *Thra2* (C), and *Ar* (D) were measured using qPCR in control and decaBDE-exposed isolated Sertoli cells cultured for 1 week. Transcript expression was normalized to the level of *Actb* transcript expression and is shown as the ratio relative to *Actb* compared with the control (set as a value of 1.0). Each value is the mean ± SD of 6-9 samples per group. *P<0.05 compared with the control.

We confirmed that THRA is expressed in Sertoli cells (**Fig. S1**). Other studies have shown that transcripts of *Thra1* and *Thra2* are also expressed in Sertoli cells [29]. The effect of postnatal exposure to decaBDE on expression of the *Thra* gene and its splicing variants (*Thra1* and *Thra2*) in Sertoli cells was evaluated by determining the levels of respective transcripts using qPCR with cDNAs obtained from isolated Sertoli cells. The level of *Thra* transcripts was significantly lower in mice exposed to decaBDE at 0.025 mg/kg than in controls (Fig. 3A), whereas no significant differences were observed at higher decaBDE doses. The level of *Thra1* transcripts was significantly lower in mice exposed to decaBDE at 0.025 mg/kg, whereas no significant differences were observed at higher decaBDE doses (Fig. 3B). The level of *Thra2* transcripts was slightly higher in decaBDE-exposed mice compared with controls; however, the differences were not significant (Fig. 3C).

### Transcript levels of *Ar* were decreased in isolated Sertoli cells of mice exposed to 0.025 mg decaBDE/kg

As T_3_ reportedly upregulates transcription of *Ar* in Sertoli cells *in vitro* [23], we measured the level of *Ar* transcripts to assess the effect of decaBDE exposure on expression of this gene. Interestingly, the level of *Ar* transcripts in isolated Sertoli cells exposed to decaBDE at 0.025 mg/kg was significantly lower than in controls (Fig. 3D). Although the level of *Ar* transcripts increased slightly in mice exposed to decaBDE at 0.25 and 2.5 mg/kg, the differences were not significant between the control and the dose groups.

### Expression levels of *Hnrnpa1* and *Srsf1* were increased in isolated Sertoli cells of mice exposed to 0.25 mg decaBDE/kg

**Figure 4:**
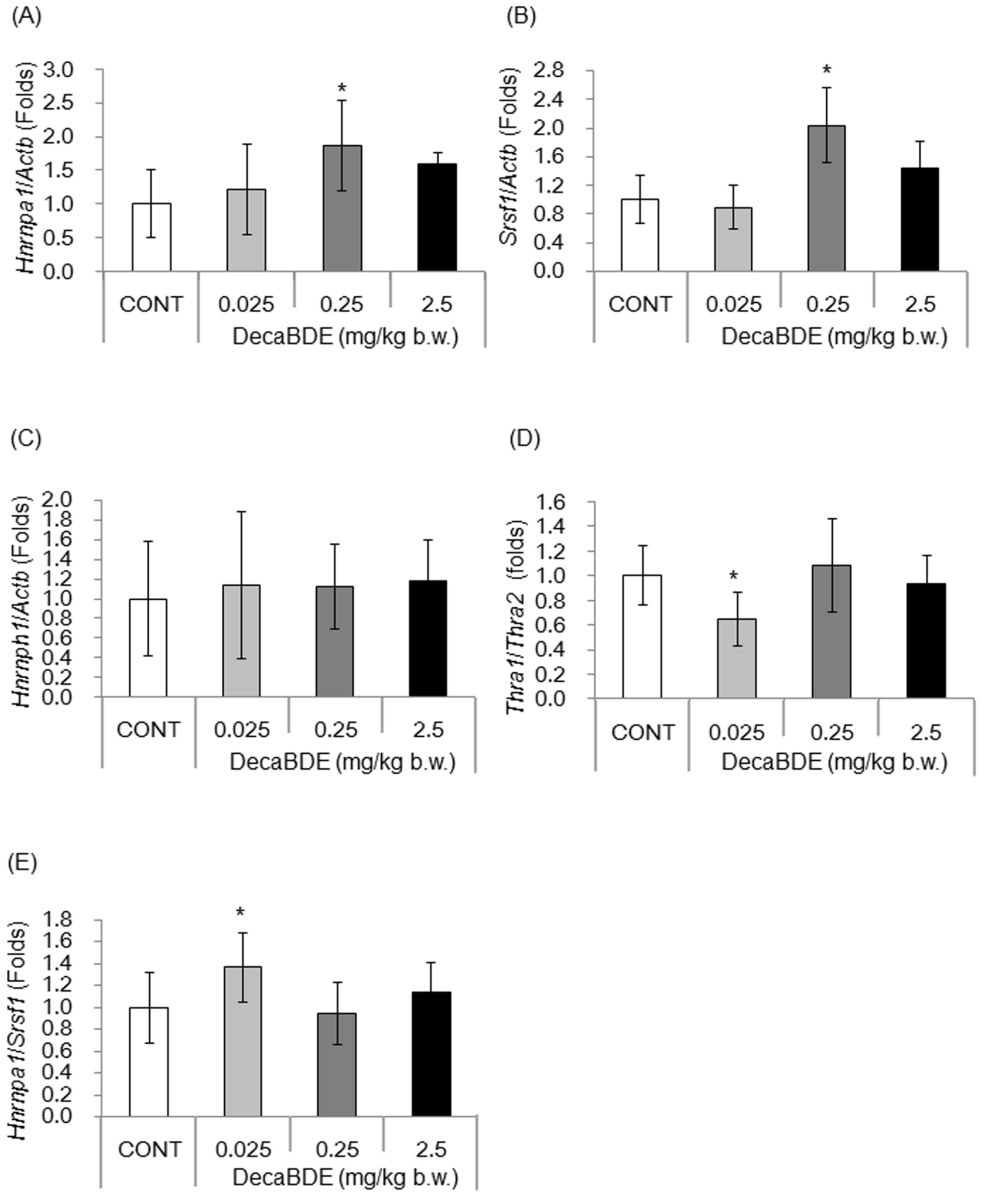
Levels of splicing factor transcripts in isolated Sertoli cells. Levels of transcripts of *Hnrnpa1* (A), *Srsf1* (B), and *Hnrnph1* (C), the *Thra1*:*Thra2* transcript expression ratio (D), and the *Hnrnpa1*:*Srsf1* transcript expression ratio (E) were measured using qPCR in isolated Sertoli cells cultured for 1 week. Each value is normalized to the transcript level of *Actb* and is shown as the ratio relative to the control (set as a value of 1.0). Each value is the mean ± SD of 6–9 samples per group. *P<0.05 compared with the control.

We confirmed that the *Thra2* splicing factors HNRNPA1, SRSF1, and HNRNPH1 were expressed in Sertoli cells (**Fig. S2**) and thus were available to mediate splicing of the *Thra* gene. The effect of postnatal exposure to decaBDE on regulation of *Thra* splicing in isolated Sertoli cells was evaluated by measuring the levels of *Hnrnpa1*and *Srsf1* transcripts. The levels of *Hnrnpa1* and *Srsf1* transcripts were significantly higher in mice exposed to decaBDE at 0.25 mg/kg than in the controls (Fig. 4A, B). No significant differences in the levels of *Hnrnpa1*and *Srsf1* transcripts were observed at lower and higher decaBDE dose groups (Fig. 4A, B). Exposure to decaBDE had no effect on *Hnrnph1* expression between the control and dose groups (Fig. 4C).

### Expression ratios of *Thra1:Thra2* and *Hnrnpa1:Srsf1* were oppositely altered in isolated Sertoli cells of mice exposed to 0.025 mg decaBDE/kg

The *Thra1*:*Thra2* expression ratio was significantly lower in Sertoli cells of mice exposed to decaBDE at 0.025 mg/kg than in controls (Fig. 4D), whereas no significant differences were observed at higher decaBDE dose groups (Fig. 4D).

The *Hnrnpa1*:*Srsf1* expression ratio was significantly higher in Sertoli cells of mice exposed to 0.025 mg decaBDE/kg than in controls (Fig. 4E), whereas no significant differences were observed at higher decaBDE dose groups. Thus, exposure to a low (0.025 mg/kg) dose of decaBDE had opposite effects on the *Thra1*:*Thra2* and *Hnrnpa1*:*Srsf1* expression ratios in isolated Sertoli cells.

## Discussion

Our previous study reported that early postnatal exposure to a low dose (0.025 mg/kg) of decaBDE has adverse effects in male mice: reduced testicular weight, lower numbers of Sertoli cells and elongated spermatids, and reduced sperm count [11]. However, these changes were not observed in the high dose (2.5 mg/kg) decaBDE-exposed group. These findings suggest that different mechanism may be involved following postnatal exposure to low and high doses of decaBDE.

Although we hypothesized that postnatal exposure to decaBDE may disrupt normal serum TH levels due to similarities in chemical structure [1]–[3], we found that postnatal exposure to decaBDE from PND 1 through 5 had no impact on serum TH levels (Fig. 1). In mice, development of the thyroid glands begins 8.5–10 days postconception appears 17.5 days postconception [30], [31]. The period of decaBDE administration in this study did not encompass the window of thyroid gland development, which could explain why we observed no effect on serum TH levels.

In contrast to serum THs, exposure to decaBDE at 0.025 or 0.25 mg/kg resulted in a significant reduction in serum testosterone levels (Fig. 2). This finding is consistent with that of another study [9] involving a different decaBDE dose. In addition, the level of *Ar* transcripts in isolated Sertoli cells declined significantly following exposure to decaBDE 0.025 mg/kg (Fig. 3E). Testosterone is important for normal spermatogenesis and development of male reproductive organs, acting on target cells via the AR [32], [33]. The phenotype of Sertoli cell-specific *Ar* gene-deletion mice includes smaller testicular size and no other male reproductive organs (seminal vesicles, prostate, or epididymis) [21]. A previous study showed reduced testicular weight and reduced numbers of Sertoli cells, elongated spermatids, and sperm in mice exposed postnatally to decaBDE at 0.025 mg/kg but no such effects in mice exposed to decaBDE at 0.25 or 2.5 mg/kg [11]. Although we did not examine steroidogenesis or Leydig cell function in the present study, the observed decreases in the levels of serum testosterone and *Ar* transcripts in Sertoli cells could cause reductions in testicular size and number of sperm in mice dosed postnatally with decaBDE at 0.025 mg/kg. Further studies are necessary to elucidate the mechanism underlying the observed reductions in testosterone and *Ar* transcript levels following postnatal exposure to decaBDE at 0.025 mg/kg.

We found that the levels of *Thra* and *Thra1* transcripts in isolated Sertoli cells (Fig. 3A, B) decreased significantly following exposure to decaBDE at 0.025 mg/kg. However, the exposure to decaBDE at 0.025 mg/kg had no significant effect on the levels of *Thra2*, *Hnrnph1*, or *Srsf1* transcripts in isolated Sertoli cells (Fig. 3C and Fig. 4B, C). These findings suggest that postnatal exposure to decaBDE on PNDs 1 through 5 has no impact on *Thra2* splicing. At present, we cannot explain the mechanism underlying the down-regulation of *Thra* and *Thra1* expression induced by exposure to the lower dose of decaBDE. It is possible that the *Thra1* and *Thra2* splicing processes differ. In mice, Sertoli cells develop in the fetal period and end by PND 15 [34], [35]. As the administration period of this study encompassed PNDs 1 through 5, the decaBDE likely affected the development of Sertoli cells, including the splicing process of *Thra* variants. Moreover, both THRA and AR are members of the nuclear receptor superfamily and are known to be expressed in Sertoli cells [36]. The present study found that expression of the *Thra* and *Ar* genes decreased significantly following exposure to decaBDE at 0.025 mg/kg. Although some studies have examined the effect of THs on expression of *Thra* and *Ar* genes [23], [37], there are no studies have examined the functional relationship between THRA and AR in Sertoli cells. Further research is needed to elucidate the detail mechanism following early postnatal exposure to decaBDE at 0.025 mg/kg on down-regulation of *Thra* and *Ar* genes in Sertoli cells.

Exposure to decaBDE at 0.025 mg/kg disturbed the normal *Thra1*:*Thra2* and *Hnrnpa1*:*Srsf1* expression ratios in isolated Sertoli cells, whereas exposure to higher doses had no significant effect (Fig. 4D, E). Moreover, exposure to the low dose of decaBDE had opposite effects on the *Thra1*:*Thra2* and *Hnrnpa1*:*Srsf1* expression ratios in isolated Sertoli cells, consistent with the results of other studies [28], [38]. The *Thra1*:*Thra2* expression ratio varies based on the tissue or cell type and is affected by changes in physiological conditions, such as may result from fasting [38]–[40]. Our study is the first to demonstrate that chemical exposure may also cause imbalances in the normal *Thra1*:*Thra2* and *Hnrnpa1*:*Srsf1* expression ratios, suggesting that these ratios may be useful as markers of drug-associated toxicity.

**Figure 5:**
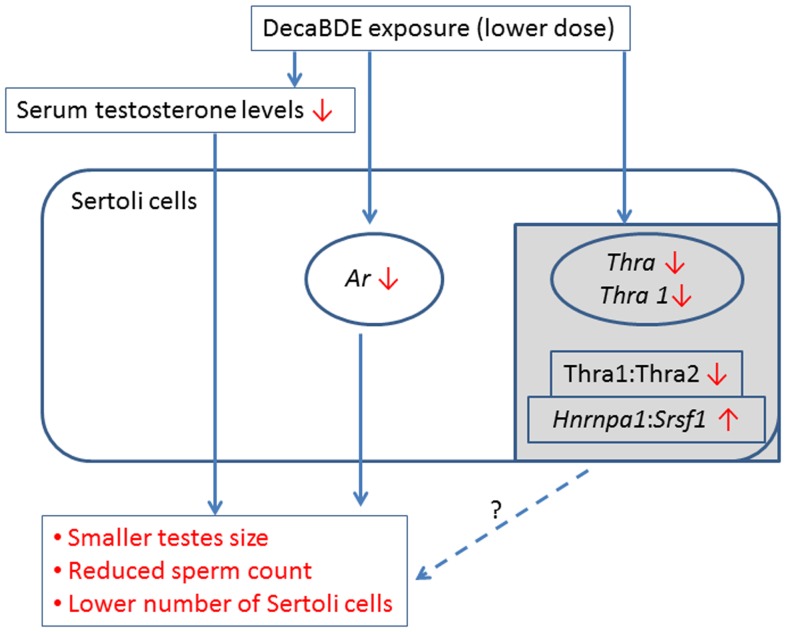
Possible mechanism following early postnatal exposure to a low dose of decaBDE (0.025 mg/kg) in mouse testis. Early postnatal exposure to decaBDE from PND 1 to 5 causes to reduce serum testosterone level and *Ar* transcript levels in Sertoli cells, resulting in smaller testes size, reduced sperm count and a lower number of Sertoli cells, as well as decreased transcript levels of *Thra* and its splicing variant, *Thra1,* and an imbalance in the expression ratios of *Thra1*:*Thra2* and *Hnrnpa1*:*Srsf1* (gray box). It remains unknown if these latter changes are related to smaller testes size, reduced sperm count and lower number of Sertoli cells (dotted line).

In conclusion, we found that postnatal exposure to a low concentration (0.025 mg/kg) of decaBDE results in significant reductions in the levels of serum testosterone and transcripts of *Ar*, *Thra*, and its variant, *Thra1* in isolated Sertoli cells (Fig. 5). The same dose leads to significant alterations in the normal *Thra1*:*Thra2* and *Hnrnpa1*:*Srsf1* expression ratios, whereas higher decaBDE doses of 0.25 and 2.5 mg/kg have no significant effect on these ratios. Our results suggest that the above-mentioned changes resulting from postnatal exposure to a low dose of decaBDE on PNDs 1 through 5 are sufficient to cause reduced testicular size and reduced numbers of Sertoli cells and sperm. Further research is necessary to more fully elucidate the detailed mechanism(s) of the toxicity associated with exposure to low decaBDE doses between PND 1 and 5 in both Ledydig and Sertoli cells.

## Supporting Information

Figure S1 **Expression and localization of THRA in mouse testes.** Sections of adult mouse testes were immunostained with antibodies to THRA (A) and the Sertoli cell marker GATA4 (B). Insets show higher magnification. Yellow indicates colocalization of THRA and GATA4 (C). Bars = 50 µm.
(TIF)

Figure S2 **Expression and localization of HNRNPA1, SRSF1, and HNRNPH1 in mouse testes.** Sections of adult mouse testes were double-immunostained with antibodies to HNRNPA1 (A), SRSF1 (D), HNRNPH (G) and the Sertoli cell marker GATA4 (middle panels, B, E, H). Insets show higher magnification. Yellow indicates colocalization of HNRNPA1, SRSF1, or HNRNPH and GATA4 (C, F, I). Bars = 50 µm.
(TIF)
