# Supplementary figures and images for: Early Postnatal Exposure to a Low Dose of Decabromodiphenyl Ether Affects Expression of Androgen and Thyroid Hormone Receptor-Alpha and Its Splicing Variants in Mouse Sertoli Cells
Source: PLoS One. 2014 Dec 5;9(12):e114487. doi: 10.1371/journal.pone.0114487 (PMC4257688; doi:10.1371/journal.pone.0114487)

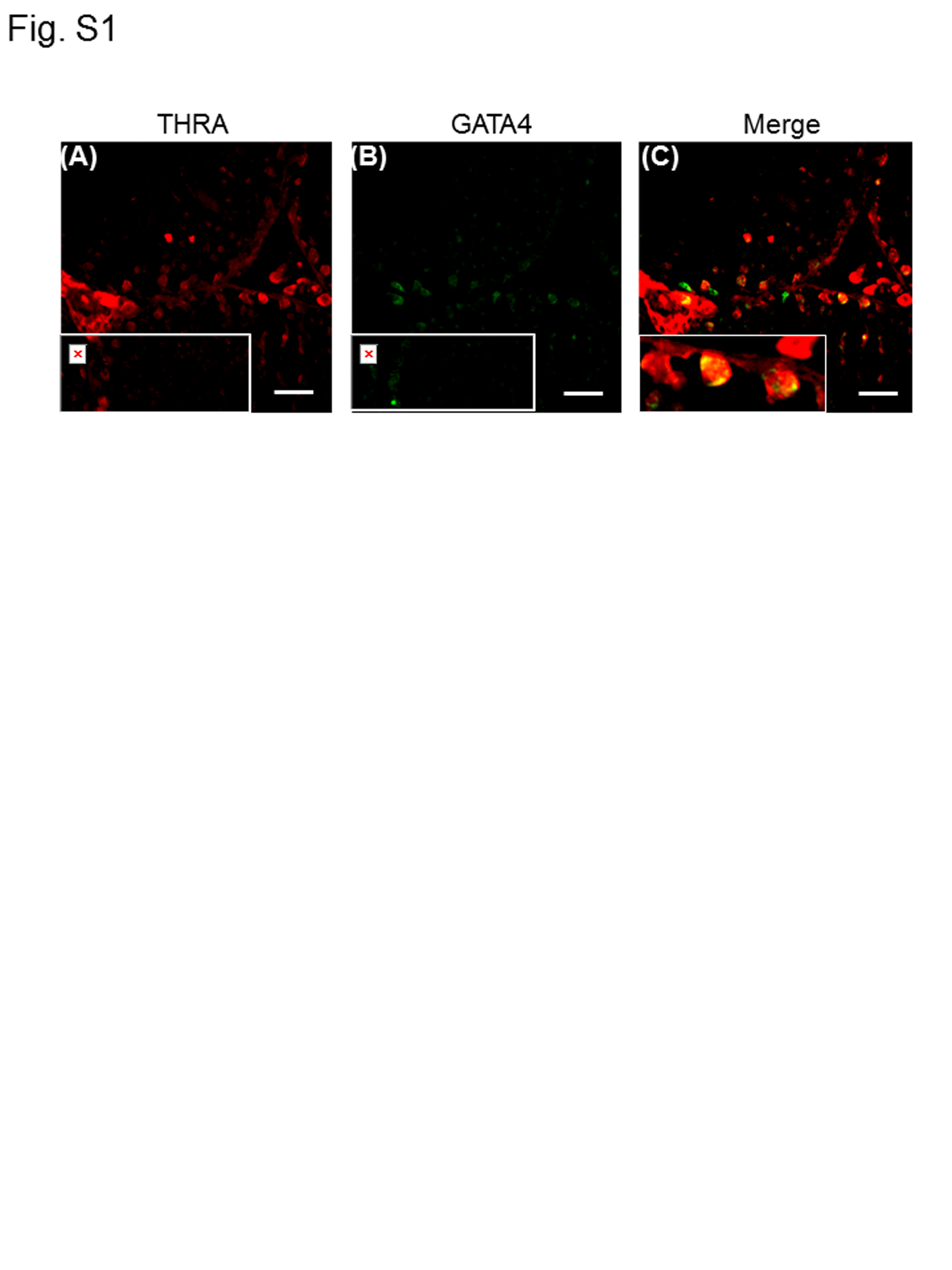

Supplement: Figure S1 — Expression and localization of THRA in mouse testes. Sections of adult mouse testes were immunostained with antibodies to THRA (A) and the Sertoli cell marker GATA4 (B). Insets show higher magnification. Yellow indicates colocalization of THRA and GATA4 (C). Bars = 50 µm. (TIF) [file pone.0114487.s001.tif]

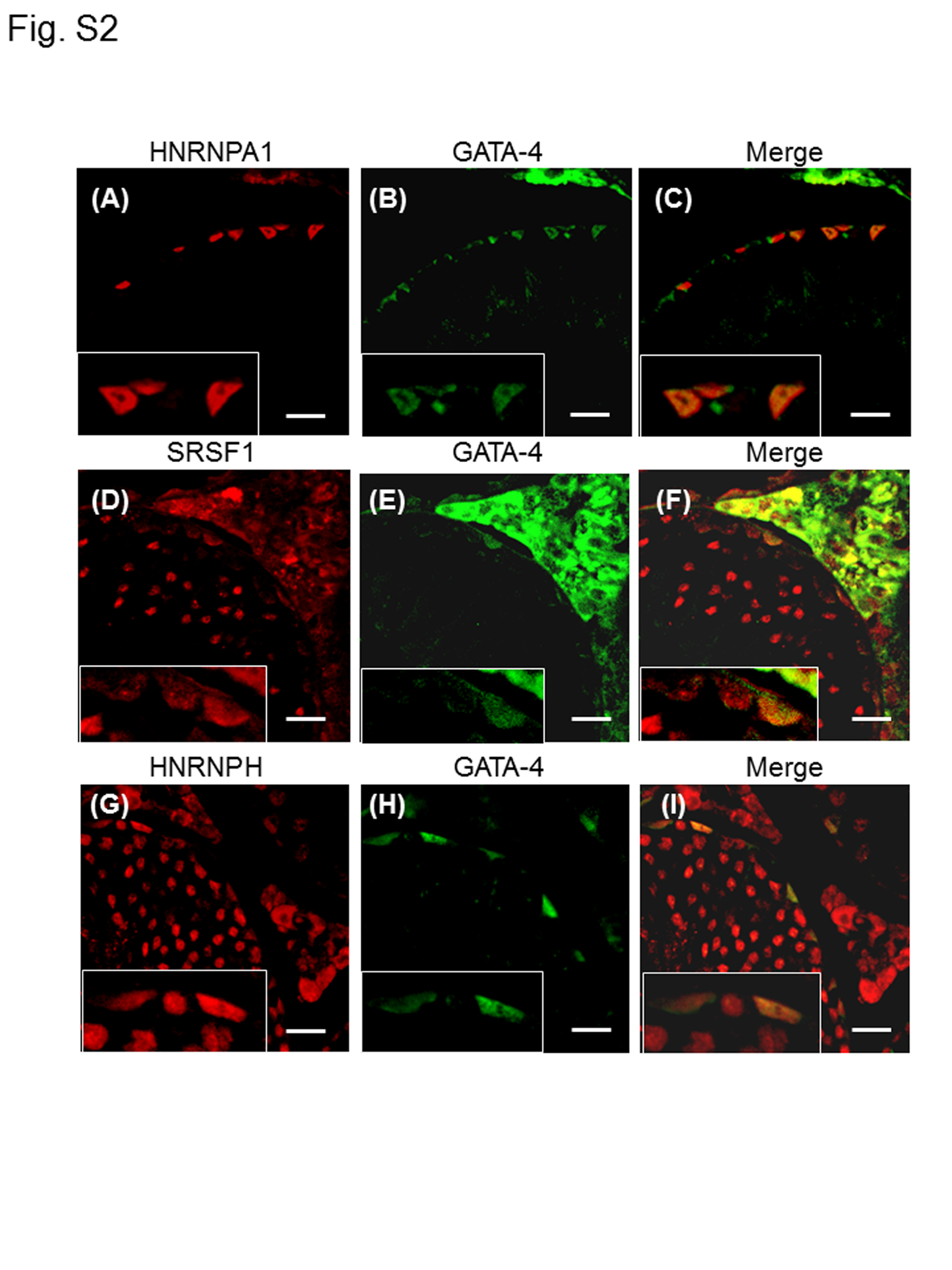

Supplement: Figure S2 — Expression and localization of HNRNPA1, SRSF1, and HNRNPH1 in mouse testes. Sections of adult mouse testes were double-immunostained with antibodies to HNRNPA1 (A), SRSF1 (D), HNRNPH (G) and the Sertoli cell marker GATA4 (middle panels, B, E, H). Insets show higher magnification. Yellow indicates colocalization of HNRNPA1, SRSF1, or HNRNPH and GATA4 (C, F, I). Bars = 50 µm. (TIF) [file pone.0114487.s002.tif]
